# Impact of a complex health services intervention in long-term care nursing homes on 3-year overall survival: results from the CoCare study

**DOI:** 10.1186/s12913-024-10635-7

**Published:** 2024-02-14

**Authors:** Klaus Kaier, Boris A. Brühmann, Stefan Fetzer, Rieka von der Warth, Erik Farin-Glattacker

**Affiliations:** 1 Institute of Medical Biometry and Statistics, Faculty of Medicine and Medical Center, University of Freiburg, Hugstetter Str. 49, 79106 Freiburg, Germany; 2 Section of Health Care Research and Rehabilitation Research (SEVERA), Institute of Medical Biometry and Statistics, Faculty of Medicine and Medical Center, University of Freiburg, Freiburg, Germany; 3 Faculty of Economics, Aalen University, Aalen, Germany

**Keywords:** Coordinated medical care, Long-term care homes, Computerized documentation system, Hospital admissions, Complex intervention, Secondary data, Claims data

## Abstract

**Background:**

The Coordinated medical Care (CoCare) project aimed to improve the quality of medical care in nursing homes by optimizing collaboration between nurses and physicians. We analyze the impact of the CoCare intervention on overall survival.

**Methods:**

The effect of time-varying treatment on 3-year overall survival was analyzed with treatment as time-varying covariate within the entire cohort. To reduce bias due to non-random assignment to treatment groups, regression adjustment was applied. Therefore, age, sex, and level of care were used as potential confounders.

**Results:**

The study population consisted of 8,893 nursing home residents (NHRs), of which 1,330 participated in the CoCare intervention. The three-year overall survival was 49.8% in the entire cohort. NHRs receiving the intervention were associated with a higher survival probability compared to NHRs of the control group. In a univariable cox model with time-dependent treatment, the intervention was associated with a hazard ratio of 0.70 [95%CI 0.56–0.87, *p* = 0.002]. After adjustment for age, sex and level of care, the hazard ratio increased to 0.82 but was still significant [95%CI 0.71–0.96, *p* = 0.011].

**Conclusion:**

The analysis shows that optimizing collaboration between nurses and physicians leads to better survival of NHRs in Germany. This adds to the already published favorable cost-benefit ratio of the CoCare intervention and shows that a routine implementation of optimized collaboration between nurses and physicians is highly recommended.

**Supplementary Information:**

The online version contains supplementary material available at 10.1186/s12913-024-10635-7.

## Introduction

In the last three decades, the number of people cared for in nursing homes (NHs) has risen sharply. Almost 800,000 people are currently receiving full-time inpatient care [1]. Many nursing home residents (NHRs) are multimorbid, have complex care needs and take multiple medications [2–6]. This, combined with a shortage of qualified staff, makes it increasingly difficult to provide adequate medical care. The care provided in nursing homes is already generally perceived as inadequate [7–13].

Underuse and misuse of medical services among NHRs have been linked to a shortage of interprofessional cooperation, communication and documentation between doctors and nurses [14–16]. In particular, nurse preparedness and physician attitudes (e.g. professionalism, responsiveness) were found to be of paramount importance for interprofessional communication [17]. Other important factors for successful cooperation between GPs and nurses are mutual trust, more contacts, fixed agreements and regular rounds [18]. A change of the resident’s family GP to reduce the number of GPs providing care may also be useful, as well as a link to an outpatient clinic in the absence of specialist care [19].

However, implementation of the necessary measures is often hampered by inadequate infrastructure and lack of or poor communication between physicians and nurses [20, 21]. In Germany, NHs have to organize cooperation with general practitioners (GPs) and specialists themselves, which leads to a high level of bureaucracy in NHs and not to a more efficient distribution of resources [22]. Compared to other countries, this is a very German specific problem. While German GPs may visit NHs as part of their main work, countries like the Netherlands and France introduced the specialization of a nursing home GP [23]. In addition, the institutional separation of health and long-term care insurance due to interface problems leads to increased under- and inappropriate supply for care for NHRs [24]. From the perspective of health and long-term insurance funds, the institutional separation of health and long-term care insurance leads to incentives to shift costs from one side to the other.

The Coordinated Medical Care (CoCare) project is designed to improve the coordination of medical care in German long-term care hospitals by optimising collaboration between nurses and physicians. The present study examines the impact of the CoCare intervention on 3-year survival among the participants of the study. This prospective, nonrandomized study is based on German insurance data, and includes a total of 8,893 residents in NHs, of which 1,330 participated in the CoCare intervention.

## Methods

A detailed overview of the study was published previously as a study protocol [10], as well as the results of the economic evaluation of CoCare [7].

Briefly, the CoCare intervention included the following elements: A team of GPs provided care to NHRs. The assignment of nursing homes was non-random. As described in more detail before [7], the intervention was administered in 35 NHs in the administrative districts of Stuttgart, Karlsruhe, and Freiburg. The 280 NHs forming the control group were recruited from another administrative district (Tübingen), which was chosen to mirror the intervention districts in number of physicians, inhabitants, and similarity in counties. GPs were allowed to treat any patient on behalf of another GP and were available by telephone out of medical treatment hours. Specialists made regular visits, at least quarterly, coordinated by GPs and accompanied by nursing staff. A coordinated medication management was implemented. Medication plans were written by GPs and reviewed quarterly. A project-specific remuneration plan includes a number of additional billing options for the participating physicians and thus significantly weakens the ceiling on costs that prevails in the German outpatient sector. For example, a project-specific surcharge was paid for joint nurse GP patient visits (€10) and joint nurse specialist patient visits (€15) or medication checks with a coordinated medication management (€10). In addition, interpatient activities such as telephone standby outside normal practice hours (€50) were remunerated separately. Communication and cooperation between physicians and nurses was improved through the appointment of study coordinators (“CoCare coordinators”) at each participating NH. CoCare coordinators were in charge of tasks, such as documentation, preparation, and follow-up of onsite physician visits. In addition, structured processes between physicians and nurses were facilitated, such as standard operating procedures for unplanned events (e.g. crisis management). Finally, treatment procedures (e.g. regarding pain) were structured and developed for participating specialists and GPs.

Details regarding the selection of intervention group NHs and control group NHs were published previously [7, 10]. Briefly, all participants in the present study had to be in a NH at least 90 days before inclusion. Dementia was an exclusion criterion. In addition, all patients that met the inclusion criteria and resided in an intervention group NH were offered to participate, provided they signed an informed consent. Patients living in a control group NH did not need to provide informed consent.

April 1st 2017 was defined as day 0 in the survival analyses. Follow-up regarding overall survival was available until April 30 2020 for the entire study population using information from participating health insurance funds. Start of the intervention (the day when informed consent was provided) took place between October 2017 and March 2020).

All statistical analyses were performed with Stata 17 (StataCorp, Texas, USA). Between-group differences among baseline characteristics were analyzed using unpaired t-test and chi-squared test. In order to outline 3-year survival within different subgroups, the Kaplan-Meier method was applied to calculate 3-year survival probabilities and median survival. In subgroups where median survival times were not measurable, parametric survival analyses with a Weibull distribution were applied to estimate median survival. The effect of time-varying treatment on mortality was analyzed with intervention as time-varying covariate within the entire cohort. To reduce bias due to non-random assignment to intervention groups, regression adjustment was applied. Therefore, age, sex and level of care were used as potential confounders. In Germany, the level of care may be interpreted as the level of dependency. There are different levels of care, which determine, among other things, how much financial support a patient receives from the statutory long-term care insurance. To determine level of care, an assessment is carried out, which evaluates the individual’s ability to perform everyday activities and the level of support required. As a higher level of care translates into more financial support from the statutory long-term care insurance, the level of care may change frequently over time when the degree of care dependency increases. Therefore, we used the latest level of care at the beginning of the observation period (April 1st 2017) as a time-fixed covariate. The regression model chosen was a cox regression model in which the multilevel structure of the data was taken into account by specifying cluster-robust standard errors at the NH level. To graphically illustrate the impact of the time-varying treatment on overall survival, the Simon–Makuch method was applied with a 12- and a 21 months landmark [25]. A 12 month landmark may be seen as the lower bound of potential landmarks, as roughly 10% of patients in the intervention group already started the intervention at month 12. Month 21, on the contrary, represents the median time to intervention which is usually chosen [26, 27]. To outline the effect of treatment over time, we employed the flexible parametric regression model, *stpm2* command in Stata, where the hazard ratio was estimated and plotted as a function of time [28]. To identify potential subgroup effects, Cox regression models with interaction between time-varying treatment and sex, age, or levels of care were specified. For age and levels of care, restricted cubic splines were used to model the non-linear interaction between the intervention and age (or level of care). Then, the *partpred* command in Stata was used to obtain estimates of the hazard ratio for the intervention as a function of age (or level of care) [29].

## Results

The characteristics of the study population (*N* = 8,893) are shown in Table 1. NHRs of the intervention group (*N* = 1,330) were younger (*p* < 0.001), more often male (*p* = 0.004), and had a lower level of care compared to NHRs of the control group (see Table 1 for details). On average, the start of the intervention was 20 months after the start of the study period. See Figure S1 for a visualization of the timing of the introduction of the intervention.

**Table 1:** Baseline characteristics

	Al Patients		Intervention		Controls		*P*-value^*^
	8,893		1,330		7,563		
Female Sex	70.73%		67.44%		71.31%		0.004
Age	83.09	10.84	79.01	11.68	83.81	10.52	< 0.001
< 65	6.34%		13.01%		5.17%		< 0.001
65–75	7.01%		13.01%		5.95%		
75–85	34.51%		37.67%		33.95%		
85–95	44.08%		32.48%		46.12%		
≥ 95	8.06%		3.83%		8.81%		
Level of care							
0–1	12.16%		23.91%		10.09%		< 0.001
2	16.87%		16.99%		16.85%		
3	27.03%		24.21%		27.53%		
4	29.03%		24.51%		29.83%		
5	14.91%		10.38%		15.71%		

^*^ Sex, age (categorical) and level of care were compared using Chi squared tests. Age (continuous) was compared using a *t*-test

**Fig. 1:**
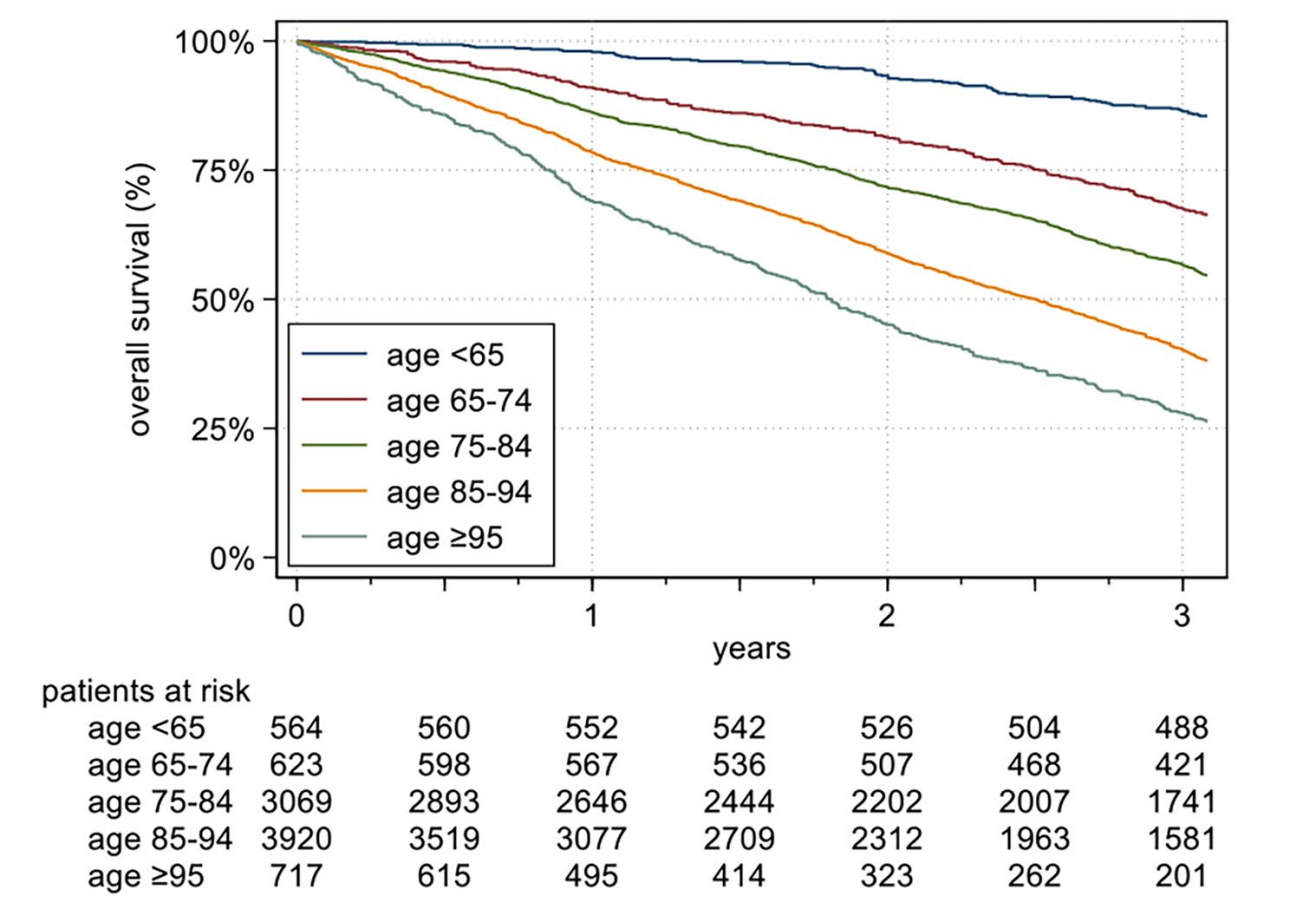
Kaplan-Meier plot of overall survival according to age

The three-year overall survival was 49.77% [95%CI 48.73-50.80%] in the entire cohort. Looking at the different age groups, (expected) differences in survival rates emerge: While 86% of patients aged < 65 were still alive after three years, the 3-year survival rate in the oldest group was only 28% (see Fig. 1). Detailed survival probabilities and median survival times can be found in Table S1 and Figure S2. Regarding the care status, a similar picture emerges: Patients with a care level of 0 or 1 have a survival probability of 86% and a median survival time of more than 4 years. For patients with a higher care level, the probability of survival decreases accordingly to a median survival time of only 2 years for patients with care level 5. Interestingly, male and female patients had comparable survival probabilities over the entire study period (Table S1). Looking at the survival probabilities of the sexes within the age groups separately, however, a completely different picture emerges. As can be seen in Table S2, women, as expected, have higher survival probabilities and longer median survival times when compared to men at the same age. Since the proportion of women increases disproportionately in the older age groups, the age difference is not visible when simply looking at the cohort as a whole (Table S1).

NHRs of the intervention group were associated with a higher survival probability compared to NHRs of the control group. In a univariable cox model with time-dependent treatment, the intervention was associated with a hazard ratio of 0.70 [95%CI 0.56–0.87, *p* = 0.002]. After adjustment for age, sex and level of care, the hazard ratio decreased to 0.82 but was still significant [95%CI 0.71–0.96, *p* = 0.011]. As shown in Table 2, male sex, high age and a higher level of care were associated with inferior survival in our multivariable cox regression model.

**Table 2:** Results of the multivariable cox regression model (*N* = 8,893)

	HR	*P*-Value	95%CI	
Intervention	0.82	0.011	0.71	0.96
Female Sex	0.72	< 0.001	0.67	0.77
Age				
< 65	1 (ref.)			
65–75	2.66	< 0.001	1.98	3.59
75–85	4.15	< 0.001	3.11	5.55
85–95	6.82	< 0.001	5.11	9.09
≥ 95	9.75	< 0.001	7.24	13.14
Level of care				
0–1	1 (ref.)			
2	1.35	0.002	1.11	1.62
3	1.18	0.103	0.97	1.45
4	1.65	< 0.001	1.35	2.01
5	2.06	< 0.001	1.68	2.53

**Fig. 2:**
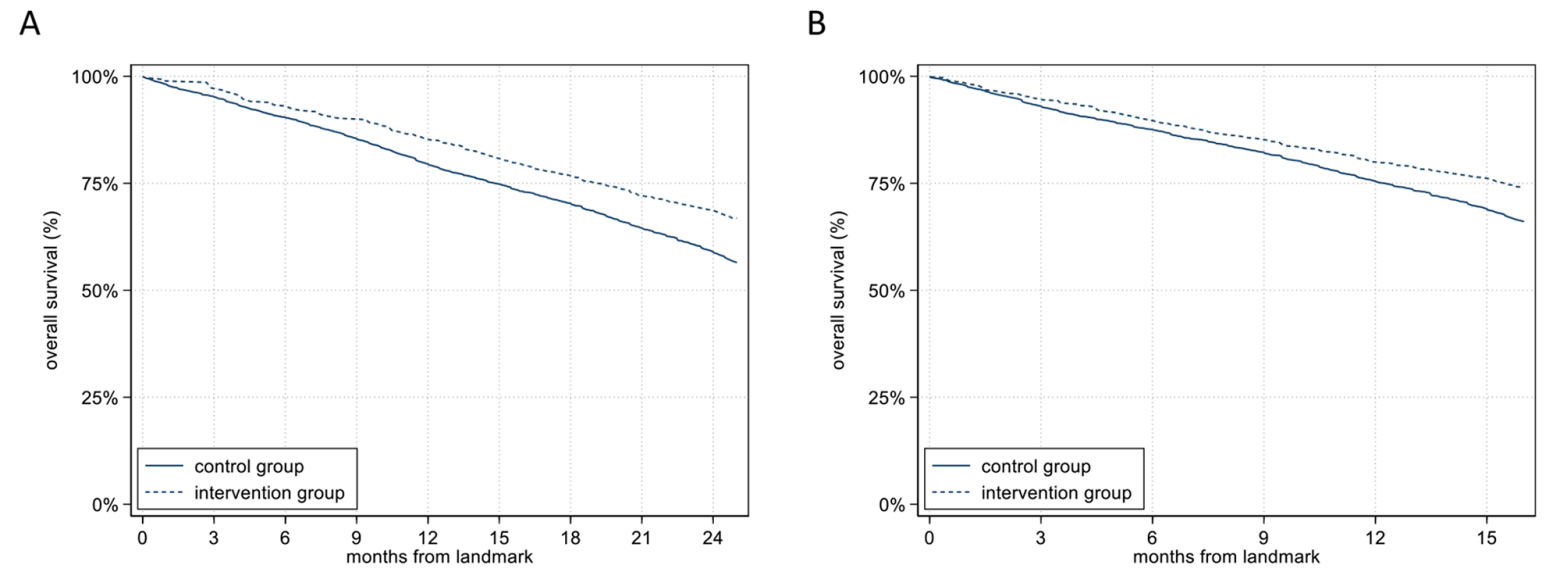
Simon–Makuch plots with landmark periods of 12-months (**A**) and 21-months (**B**)

As shown in Fig. 2, Simon–Makuch plots illustrate the steadily higher survival probabilities of the NHRs of the intervention group compared to those of the control group.

Interactions between intervention and the confounders were tested but found insignificant (*p* = 0.181, *p* = 0.226, *p* = 0.320 for sex, age and level of care, respectively). Although no significant interaction was found, visualizations of the nonlinear relationship between age or level of care and the treatment effects are shown in Figure S3. Regarding the interaction with sex, female NHRs [HR 0.78, 95%CI 0.64–0.95, *p* = 0.014] tend to benefit more from the intervention than male NHRs [HR 0.93, 95%CI 0.78–1.09, *p* = 0.358]. Inspection of the effect of the intervention over time shows no relevant trend (see Figure S4).

## Discussion

The analysis shows that the CoCare intervention leads to better survival of NHRs in Germany. Previous analyses have already shown the favorable cost-benefit ratio of the intervention with respect to healthcare costs. Specifically, the total cost of medical service use was reduced by €468.56 (*p* < 0.001) per NHR per quarter [7], meaning that the benefit of the intervention – avoided hospital admissions – clearly exceeded the cost of additional outpatient billings [see [7] for details]. When a new intervention is superior in both outcomes and cost savings, it is called an economically “dominant” strategy. In fact, very few interventions fall into this category; the most common scenario is that a new strategy improves outcomes at a higher cost. Therefore, the routine implementation of optimized collaboration between nurses and physicians is highly recommended.

In view of the substantial effects of the intervention, the individual components of the intervention are to be discussed in order to enable at least partial implementation of what saves lives and reduces costs.

From the perspective of NHRs, the CoCare intervention provided above all more services. In general, NHRs have a high prevalence of a variety of health problems [30]. Particularly needed are physiotherapists (91%), psychiatrists/neurologists (89.3%), dentists (73.7%) and urologists (71.3%) [31], whose lack causes health problems and increased hospitalizations. With a billable service catalogue for physicians and treatment guidelines for nurses, the CoCare intervention started here. Furthermore, the hurdle for NHs to contact GPs was greatly reduced in CoCare, because of their increased availability, regular visits and meetings as well as indication-specific case conferences. This ensured that GPs were often contacted at an early stage, or even in unclear situations.

From the perspective of nurses, the CoCare intervention meant more knowledge and skills to do the right thing at the right moment. Nurses are the first point of medical contact for NHRs. This is often an excessive demand in terms of content [32]. Therefore, treatment pathways were developed for the CoCare intervention (pain, challenging behavior, polypharmacy and transition from curative to palliative care) and were distributed among nursing staff. A subsidized CoCare coordinator in each NH was in charge of documentation, preparation and follow up of on-site physician visits, which lead to more productive meetings.

From the perspective of the treating GPs, the CoCare intervention primarily meant more direct and closer contact with patients and stronger networking in the NHs. This is particularly evident in the example of polypharmacy. The potentially inappropriate use of medications is common, avoidable and often associated with negative consequences for patients [33]. Therefore, competent and careful medication management is required for complex medication care. In the CoCare intervention, this was carried out jointly by GPs and nursing staff at regular intervals during joint ward rounds. The formation of teams of physicians helped to stand-in for other GPs and allocate NH visits. Last but not least, the project-specific remuneration plan includes a number of additional billing options for participating physicians and thus significantly weakens the ceiling on costs that prevails in the German outpatient sector. We believe that the project-specific surcharges have been instrumental in improving the intensity and quality of care.

Although the study was limited to Germany, one might argue the results are also of relevance for other countries, in which similar questions are likely to arise with the ageing of the population. Especially the German institutional separation of health and long-term care insurance and it’s impact on the inappropriate supply for care for NHRs is likely to be present in a lot of European countries. According to the European Commission, only a minority of countries (Denmark, Ireland and Portugal) are organized in a way which integrates health and long-term care. In most countries, however, the institutional separation between health and long-term care is described as the same problematic vertical division of responsibilities as we observe it in Germany [34].

### Limitations

In interpreting our findings, a number of limitations have to be taken into consideration. As a major limitation, no randomization was possible, since this is a study in a real-world setting. Instead, regression adjustment was applied. Thereby, adjusted hazard ratios may be interpreted as ‘true’ intervention-related effects if all parameters that are relevant for the decision and the outcome are used for the risk adjustment. Unfortunately, there can be no guarantee that all relevant parameters are part of the model. In fact, the administrative data set lacks relevant medical information (such as comorbidities) and information regarding level of care may be incomplete and/or only available with a considerable time delay. Obviously, a considerable amount of information regarding level of care may be missing, since the number of NHRs with a level of care of 1 or not yet assigned is higher than the numbers reported in the official care statistic [1]. Unfortunately, no imputation of these potentially missing values could be conducted due to the absence of codes indicating that these information were missing. In addition, there may be additional unobserved differences between the intervention and control groups. An example of this would be general frailty. This is often neglected in studies due to the complexity of its collection. For example, if patients in the intervention group have better characteristics that have not yet been observed - such as less frailty. The analysis would tend to overestimate the effect of the intervention. A further limitation to the conclusion that the intervention reduces costs and saves lives at the same time is, that only healthcare costs were considered in the analysis [7]. Whether there are higher, lower, or neutral effects in terms of NH costs depends largely on whether the additional life expectancy gained through NHRs is spent in higher levels of care or whether the intervention also leads to a delay in the progression of care intensity. Unfortunately, the design of the intervention does not enable us to distinguish between the two influential factors (1) better nurse-physician collaboration, and (2) greater consumption of outpatient healthcare. Most of the billing options in the project-specific remuneration plan increases both (1) nurse-physician collaboration and (2) outpatient healthcare consumption at the same time. In addition, the exclusion criteria of dementia and a stay of less than three months substantially limit the external validity of the results. Dementia in particular is omnipresent in long-term inpatient care and our results do not allow us to draw conclusions about the effect of the CoCare intervention on patients with dementia. To answer this question, further research is needed. Finally, we believe that the intervention is most appropriate on the NH level. That is why the intervention was rolled out at the NH level. As individual consent was necessary to participate in the intervention, however, we cannot exclude possible spillover effects. If there were such spillover effects at the NH level, there could be a systematic underestimation of the intervention effect, since control patients also benefit indirectly from the intervention.

## Conclusion

The analysis shows that optimizing collaboration between nurses and physicians leads to better survival of NHRs in Germany. This adds to the already published favorable cost-benefit ratio of the CoCare intervention and shows that a routine implementation of optimized collaboration between nurses and physicians is highly recommended.

### Electronic supplementary material

Below is the link to the electronic supplementary material.

Supplementary material 1

## Data Availability

The datasets used and/or analysed during the current study available from the corresponding author on reasonable request.
