# Supplementary material for: Impact of a complex health services intervention in long-term care nursing homes on 3-year overall survival: results from the CoCare study
Source: BMC Health Serv Res. 2024 Feb 14;24:203. doi: 10.1186/s12913-024-10635-7 (PMC10868086; doi:10.1186/s12913-024-10635-7)
Supplement: Supplementary file 1 — Supplementary material 1 [file 12913_2024_10635_MOESM1_ESM.docx]

Table S1: Survival probabilities and median survival for subgroups

| \|  \| Survival probability \| \| \| \| Median survival \| \| \| \| \| --- \| --- \| --- \| --- \| --- \| --- \| --- \| --- \| --- \| \|  \| **month** \| **%** \| **95%-CI** \| \| **months** \| \| **95%-CI** \| \| \| all Patients \|  \|  \|  \|  \|  \|  \|  \|  \| \|  \| 12 \| 82.46% \| 81.65% \| 83.23% \|  \|  \|  \|  \| \|  \| 24 \| 65.96% \| 64.97% \| 66.94% \|  \| 35.87 \| 35.20 \| 36.43 \| \|  \| 36 \| 49.77% \| 48.73% \| 50.80% \|  \|  \|  \|  \| \| male \|  \|  \|  \|  \|  \|  \|  \|  \| \|  \| 12 \| 83.60% \| 82.12% \| 84.96% \|  \|  \|  \|  \| \|  \| 24 \| 65.89% \| 64.03% \| 67.67% \|  \| 35.03 \| 33.97 \| 36.27 \| \|  \| 36 \| 48.48% \| 46.55% \| 50.39% \|  \|  \|  \|  \| \| female \|  \|  \| 67.15% \|  \|  \|  \|  \|  \| \|  \| 12 \| 81.99% \| 81.02% \| 82.92% \|  \|  \|  \|  \| \|  \| 24 \| 65.99% \| 64.81% \| 67.15% \|  \| 36.13 \| 35.47 \| 36.70 \| \|  \| 36 \| 50.30% \| 49.06% \| 51.53% \|  \|  \|  \|  \| \| age <65 \|  \|  \|  \|  \|  \|  \|  \|  \| \|  \| 12 \| 97.87% \| 96.28% \| 98.79% \|  \|  \|  \|  \| \|  \| 24 \| 93.26% \| 90.86% \| 95.05% \|  \| 126.87 \| 99.05 \| 154.69 \| \|  \| 36 \| 86.35% \| 83.23% \| 88.92% \|  \|  \|  \|  \| \| age 65-74 \|  \|  \|  \|  \|  \|  \|  \|  \| \|  \| 12 \| 91.01% \| 88.48% \| 93.01% \|  \|  \|  \|  \| \|  \| 24 \| 81.38% \| 78.10% \| 84.22% \|  \| 57.74 \| 50.14 \| 65.35 \| \|  \| 36 \| 67.58% \| 63.75% \| 71.10% \|  \|  \|  \|  \| \| age 75-84 \|  \|  \|  \|  \|  \|  \|  \|  \| \|  \| 12 \| 86.15% \| 84.88% \| 87.33% \|  \|  \|  \|  \| \|  \| 24 \| 71.65% \| 70.02% \| 73.21% \|  \| 42.05 \| 38.52 \| 45.58 \| \|  \| 36 \| 56.70% \| 54.92% \| 58.43% \|  \|  \|  \|  \| \| age 85-94 \|  \|  \|  \|  \|  \|  \|  \|  \| \|  \| 12 \| 78.44% \| 77.12% \| 79.70% \|  \|  \|  \|  \| \|  \| 24 \| 58.95% \| 57.40% \| 60.48% \|  \| 30.03 \| 28.73 \| 31.03 \| \|  \| 36 \| 40.23% \| 38.69% \| 41.76% \|  \|  \|  \|  \| \| age ≥95 \|  \|  \|  \|  \|  \|  \|  \|  \| \|  \| 12 \| 69.04% \| 65.51% \| 72.28% \|  \|  \|  \|  \| \|  \| 24 \| 45.05% \| 41.37% \| 48.65% \|  \| 21.70 \| 20.07 \| 23.43 \| \|  \| 36 \| 28.03% \| 24.79% \| 31.36% \|  \|  \|  \|  \| \| level of care 0-1 \| \|  \|  \|  \|  \|  \|  \|  \| \|  \| 12 \| 97.87% \| 96.28% \| 98.79% \|  \|  \|  \|  \| \|  \| 24 \| 93.26% \| 90.86% \| 95.05% \|  \| 52.94 \| 42.48 \| 63.41 \| \|  \| 36 \| 86.35% \| 83.23% \| 88.92% \|  \|  \|  \|  \| \| level of care 2 \| \|  \|  \|  \|  \|  \|  \|  \| \|  \| 12 \| 91.01% \| 88.48% \| 93.01% \|  \|  \|  \|  \| \|  \| 24 \| 81.38% \| 78.10% \| 84.22% \|  \| 36.34 \| 33.19 \| 39.48 \| \|  \| 36 \| 67.58% \| 63.75% \| 71.10% \|  \|  \|  \|  \| \| level of care 3 \| \|  \|  \|  \|  \|  \|  \|  \| \|  \| 12 \| 86.15% \| 84.88% \| 87.33% \|  \|  \|  \|  \| \|  \| 24 \| 71.65% \| 70.02% \| 73.21% \|  \| 41.46 \| 38.77 \| 44.15 \| \|  \| 36 \| 56.70% \| 54.92% \| 58.43% \|  \|  \|  \|  \| \| level of care 4 \| \|  \|  \|  \|  \|  \|  \|  \| \|  \| 12 \| 78.44% \| 77.12% \| 79.70% \|  \|  \|  \|  \| \|  \| 24 \| 58.95% \| 57.40% \| 60.48% \|  \| 32.23 \| 31.13 \| 33.43 \| \|  \| 36 \| 40.23% \| 38.69% \| 41.76% \|  \|  \|  \|  \| \| level of care 5 \| \|  \|  \|  \|  \|  \|  \|  \| \|  \| 12 \| 69.04% \| 65.51% \| 72.28% \|  \|  \|  \|  \| \|  \| 24 \| 45.05% \| 41.37% \| 48.65% \|  \| 28.27 \| 26.13 \| 29.77 \| \|  \| 36 \| 28.03% \| 24.79% \| 31.36% \|  \|  \|  \|  \| |  |  |  |  |  |  |  |  |
| --- | --- | --- | --- | --- | --- | --- | --- | --- | --- | --- | --- | --- | --- | --- | --- | --- | --- | --- | --- | --- | --- | --- | --- | --- | --- | --- | --- | --- | --- | --- | --- | --- | --- | --- | --- | --- | --- | --- | --- | --- | --- | --- | --- | --- | --- | --- | --- | --- | --- | --- | --- | --- | --- | --- | --- | --- | --- | --- | --- | --- | --- | --- | --- | --- | --- | --- | --- | --- | --- | --- | --- | --- | --- | --- | --- | --- | --- | --- | --- | --- | --- | --- | --- | --- | --- | --- | --- | --- | --- | --- | --- | --- | --- | --- | --- | --- | --- | --- | --- | --- | --- | --- | --- | --- | --- | --- | --- | --- | --- | --- | --- | --- | --- | --- | --- | --- | --- | --- | --- | --- | --- | --- | --- | --- | --- | --- | --- | --- | --- | --- | --- | --- | --- | --- | --- | --- | --- | --- | --- | --- | --- | --- | --- | --- | --- | --- | --- | --- | --- | --- | --- | --- | --- | --- | --- | --- | --- | --- | --- | --- | --- | --- | --- | --- | --- | --- | --- | --- | --- | --- | --- | --- | --- | --- | --- | --- | --- | --- | --- | --- | --- | --- | --- | --- | --- | --- | --- | --- | --- | --- | --- | --- | --- | --- | --- | --- | --- | --- | --- | --- | --- | --- | --- | --- | --- | --- | --- | --- | --- | --- | --- | --- | --- | --- | --- | --- | --- | --- | --- | --- | --- | --- | --- | --- | --- | --- | --- | --- | --- | --- | --- | --- | --- | --- | --- | --- | --- | --- | --- | --- | --- | --- | --- | --- | --- | --- | --- | --- | --- | --- | --- | --- | --- | --- | --- | --- | --- | --- | --- | --- | --- | --- | --- | --- | --- | --- | --- | --- | --- | --- | --- | --- | --- | --- | --- | --- | --- | --- | --- | --- | --- | --- | --- | --- | --- | --- | --- | --- | --- | --- | --- | --- | --- | --- | --- | --- | --- | --- | --- | --- | --- | --- | --- | --- | --- | --- | --- | --- | --- | --- | --- | --- | --- | --- | --- | --- | --- | --- | --- | --- | --- | --- | --- | --- | --- | --- | --- | --- | --- | --- | --- | --- | --- | --- | --- | --- | --- | --- | --- | --- | --- | --- | --- | --- | --- | --- | --- | --- | --- | --- | --- | --- | --- | --- | --- | --- | --- | --- | --- | --- | --- | --- | --- | --- | --- | --- | --- | --- | --- | --- | --- | --- | --- | --- | --- | --- | --- | --- | --- | --- | --- | --- | --- | --- | --- | --- | --- | --- | --- | --- | --- | --- | --- | --- | --- | --- | --- | --- | --- | --- | --- | --- | --- | --- | --- | --- | --- | --- | --- | --- | --- | --- | --- | --- | --- | --- | --- | --- | --- | --- | --- | --- | --- | --- | --- | --- | --- | --- | --- | --- | --- | --- | --- | --- | --- | --- | --- | --- | --- | --- | --- | --- | --- | --- | --- | --- | --- | --- | --- | --- | --- | --- | --- | --- | --- | --- | --- | --- | --- | --- | --- | --- | --- | --- | --- | --- | --- | --- | --- | --- | --- | --- | --- | --- | --- | --- | --- | --- | --- | --- | --- | --- | --- | --- | --- | --- | --- | --- | --- | --- | --- | --- | --- | --- |

Table S2: Survival probabilities and median survival for combination of subgroups

|  | | Survival probability | | | Median survival | | | |
| --- | --- | --- | --- | --- | --- | --- | --- | --- |
| **Male patients** | | **%** | **95%-CI** | | **months** | | **95%-CI** | |
| age <65 |  |  |  |  |  |  |  |  |
| N=343 | 12 | 98.25% | 96.15% | 99.21% |  |  |  |  |
|  | 24 | 92.71% | 89.40% | 95.02% |  | 114.05 | 83.38 | 144.72 |
|  | 36 | 85.42% | 81.23% | 88.75% |  |  |  |  |
| age 65-74 |  |  |  |  |  |  |  |  |
| N=349 | 12 | 89.40% | 85.67% | 92.20% |  |  |  |  |
|  | 24 | 78.80% | 74.12% | 82.73% |  | 52.65 | 44.40 | 60.91 |
|  | 36 | 65.33% | 60.08% | 70.06% |  |  |  |  |
| age 75-84 |  |  |  |  |  |  |  |  |
| N=995 | 12 | 83.32% | 80.85% | 85.49% |  |  |  |  |
|  | 24 | 66.03% | 63.00% | 68.88% |  | 34.50 | 32.17 | 36.47 |
|  | 36 | 47.54% | 44.40% | 50.60% |  |  |  |  |
| age 85-94 |  |  |  |  |  |  |  |  |
| N=829 | 12 | 76.36% | 73.31% | 79.10% |  |  |  |  |
|  | 24 | 51.27% | 47.81% | 54.61% |  | 24.90 | 23.20 | 26.67 |
|  | 36 | 29.67% | 26.60% | 32.81% |  |  |  |  |
| age ≥95 |  |  |  |  |  |  |  |  |
| N=87 | 12 | 74.71% | 64.19% | 82.56% |  |  |  |  |
|  | 24 | 45.98% | 35.29% | 56.01% |  | 21.93 | 16.93 | 27.47 |
|  | 36 | 25.29% | 16.73% | 34.74% |  |  |  |  |
|  |  |  |  |  |  |  |  |  |
|  |  |  |  |  |  |  |  |  |
| **Female patients** | | **%** | **95%-CI** | | **months** | | **95%-CI** | |
| age <65 |  |  |  |  |  |  |  |  |
| N=221 | 12 | 97.29% | 94.06% | 98.77% |  |  |  |  |
|  | 24 | 94.12% | 90.09% | 96.54% |  | 138.12 | 93.53 | 182.71 |
|  | 36 | 87.78% | 82.69% | 91.45% |  |  |  |  |
| age 65-74 |  |  |  |  |  |  |  |  |
| N=274 | 12 | 93.07% | 89.34% | 95.52% |  |  |  |  |
|  | 24 | 84.67% | 79.83% | 88.43% |  | 63.83 | 52.99 | 74.67 |
|  | 36 | 70.44% | 64.65% | 75.46% |  |  |  |  |
| age 75-84 |  |  |  |  |  |  |  |  |
| N=2074 | 12 | 87.51% | 86.01% | 88.86% |  |  |  |  |
|  | 24 | 74.35% | 72.41% | 76.17% |  | 42.05 | 38.52 | 45.58 |
|  | 36 | 61.09% | 58.95% | 63.15% |  |  |  |  |
| age 85-94 |  |  |  |  |  |  |  |  |
| N=3091 | 12 | 79.00% | 77.52% | 80.40% |  |  |  |  |
|  | 24 | 61.02% | 59.27% | 62.71% |  | 31.53 | 30.47 | 32.77 |
|  | 36 | 43.06% | 41.31% | 44.80% |  |  |  |  |
| age ≥95 |  |  |  |  |  |  |  |  |
| N=630 | 12 | 68.25% | 64.46% | 71.73% |  |  |  |  |
|  | 24 | 44.92% | 41.00% | 48.76% |  | 21.53 | 19.50 | 23.43 |
|  | 36 | 28.41% | 24.94% | 31.97% |  |  |  |  |

Figure S1:  Timing of the start of the intervention

Figure S2: Kaplan-Meier plots of overall survival

| A   | B   |
| --- | --- |
| C   | D   |

Figure S3: Visualizations of the nonlinear relationship between age (A) or level of care (B) and the treatment

| A   | B   |
| --- | --- |

To identify potential subgroup effects, Cox regression models with interaction between treatment and sex, age, or the levels of care were specified. For age and levels of care, restricted cubic splines were used to model the non-linear interaction between the intervention and age (or the level of care). Then, the *partpred* command in Stata was used to obtain estimates of the hazard ratio for the intervention as a function of age (or the level of care)

Figure S4: Visualization of the treatment effect over time

To outline the effect of treatment over time, we employed the flexible parametric regression, *stpm2* command in Stata, where the HR was estimated and plotted as a function of time.
